# Are Patellofemoral Ligaments and Retinacula Distinct Structures of the Knee Joint? An Anatomic, Histological and Magnetic Resonance Imaging Study

**DOI:** 10.3390/ijerph19031110

**Published:** 2022-01-19

**Authors:** Carlo Biz, Carla Stecco, Alberto Crimì, Carmelo Pirri, Michele Fosser, Caterina Fede, Chenglei Fan, Pietro Ruggieri, Raffaele De Caro

**Affiliations:** 1 Orthopaedics and Orthopaedic Oncology, Department of Surgery, Oncology and Gastroenterology DiSCOG, University of Padua, 35128 Padova, Italy; albe.crim@gmail.com (A.C.); michele.fosser@gmail.com (M.F.); pietro.ruggieri@unipd.it (P.R.); 2 Department of Neurosciences, Institute of Human Anatomy, University of Padua, 35121 Padova, Italy; carla.stecco@unipd.it (C.S.); carmelo.pirri@unipd.it (C.P.); caterina.fede@unipd.it (C.F.); yutianfan1218@163.com (C.F.); rdecaro@unipd.it (R.D.C.)

**Keywords:** knee anatomy, knee imaging, patellofemoral ligament, retinaculum

## Abstract

There is disagreement regarding the description of the patellofemoral ligaments (PFLs), considered by some authors as capsular thickening and by others as independent ligaments. It was hypothesised that the PFLs and retinacula are structures with different histological features. The aim of this study was to describe the stabilising structures of the patella in detail and to determine if the PFLs and retinacula are different and separable structures from a macroscopic, microscopic and imaging viewpoint. An anatomical study was performed on eight knees from five cadavers (mean age, 56.2 years; range, 35–63 years), and a histological study was conducted on specimens from nine patients having a mean age of 65 years (range 35–84 years) who had undergone surgical knee procedures. The imaging study was based on 100 MRIs (96 patients). The mean age was 46 years (range 16–88), and the study analysed the capsular-ligamentous structures. In the medial compartment, the layers and structures were as follows: superficial layer, medial retinaculum; intermediate layer, Medial Collateral Ligament (MCL), Posterior Oblique Ligament (POL) and Medial Patellofemoral Ligament (MPFL); deep layer, deep part of the MCL and joint capsule. In the lateral compartment, the layers and structures were the following: superficial layer, lateral retinaculum; intermediate layer, Lateral Collateral Ligament (LCL) and Lateral Patellofemoral Ligament (LPFL); deep layer, joint capsule. All of the knees examined presented a clearly distinguishable MPFL and LPFL separable from the capsular layer. Histological study: there was a higher density of nerve fibres in retinacula compared to ligaments (*p* = 0.0034) and a higher content of elastic fibres in retinacula (*p* < 0.0005). In imaging, there was no difference between medial and lateral retinaculum thickness (*p* > 0.05). In conclusion, both the lateral and medial compartment can be described using the three-layer scheme. PFLs and retinacula are separate structures both macroscopically and according to imaging analysis. The retinacula respond to their specific function with a higher nerve fibre content and higher number of elastic fibres compared to the ligaments.

## 1. Introduction

Anatomical descriptions of the myofascial layers of the knee have been published in the last 40 years, but the attachments, name or even existence of the various layers are ill-defined, particularly regarding the knee retinacula and the patellofemoral ligaments (PFLs). Warren and Marshall [1], in 1979, were the first to describe the medial compartment of the knee joint with the three-layer concept, with the first layer being the most superficial (medial retinaculum), the second layer being intermediate (the Medial Collateral Ligament (MCL), the Posterior Oblique Ligament (POL) and the Medial Patellofemoral Ligament (MPFL)), and the third layer being the deepest (the deep part of the MCL and the joint capsule). This description was accepted by other authors [2,3,4,5,6,7]. 

For the lateral compartment, the agreement is not univocal. There is a two-layer model [8] with a superficial layer made up of the superficial oblique retinaculum and a deep layer consisting of the deep transverse retinaculum. There is also a three-layer model [9]: a superficial layer made up of derivatives of fascia, an intermediate layer of the quadriceps apparatus, and a deep layer, made up of the joint capsule. Another source of disagreement is the nature of the PFLs, considered by some authors as capsular thickening and by other authors as independent ligaments [7,10,11,12,13,14,15,16]. The patellofemoral joint has been investigated using anatomical and MRI studies in association since the 1990s [17]. In a thorough investigation, Chhabra et al. [18] proposed a systematic imaging approach to anterior and anterolateral knee pain with MRI, defining the ligamentous structures and how to evaluate them. The MPFL has also been studied with MRI, evaluating its role in patellar instability in the case of a lesion not repaired surgically [19].

The function of PFLs and retinacula has been studied from a surgical point of view, particularly the role of the MPFL in lateral patellar dislocation and its reconstruction. One of the most reported complications after reconstruction of MPFL for patellar dislocation is loss of knee flexion and medial knee pain [20]. In up to 30% of MPFL surgical reconstructions [21], the alteration of tension in the medial compartment is attributed only to the MPFL [22]. The role of retinaculum alteration in eliciting pain in the medial compartment of the knee is not taken into consideration by the current literature.

None of the published studies evaluate the PFLs and the retinacula comprehensively from anatomical, functional and MRI points of view. Therefore, the aim of this study was to describe in detail the stabilising structures of the patella, and particularly to determine if the PFLs and retinacula are different and separable structures from macroscopic, microscopic and imaging viewpoints.

It was hypothesised that the PFLs and retinacula are structures with different histological features and function with clear imaging identifiable landmarks.

## 2. Materials and Methods

### 2.1. Macroscopic Study

Eight fresh-frozen human cadaveric knees (mean age, 56.2 years; range, 35–63 years; 4 right, 4 left; 4 males, 1 female), BMI 24.2 (range 23–27), without previous injury, tumours, surgery, infection or a history of knee arthritis were used for this study. They were obtained from the ‘Body Donation Program’ at the Institute of Human Anatomy, of our university [23]. The anatomical dissection was performed with the knee in 20 degrees of flexion and neutral rotation. The medial compartment was dissected in proximal to distal direction along the deep fascia covering the vastus medialis muscle. The lateral compartment was dissected in proximal to distal direction along the deep fascia lata covering the vastus lateralis muscle. The iliotibial band was cut proximally to the knee articular line exposing the deep structures. In the lateral and medial compartments, the insertions, thickness and length of ligaments up to the capsular layer were measured with a ruler and a calibre (resolution 0.01 cm). The length and width of the patella were also measured. 

### 2.2. Microscopic Study

Nine patients (6 males and 3 females), mean age 65 years (range 35–84 years), mean BMI 25.5 (range 24–28), for a total of 9 knees, who underwent knee surgical procedures with exposure of the ligamentous structures (4 patients had surgery for proximal tibia fracture, 3 patients for total knee replacement, 2 patients for distal femur fractures) were selected for sampling after informed consent (Ethics Committee approval AMOFA Study, Prot. Num. 3722/AO/16, approved 05.13.2016).

Samples of 1 cm × 1 cm were obtained from the retinacula and PFLs. Selection criteria: patients naïve for surgery on the knee area and possibility to obtain a 1 cm × 1 cm sample of the retinacula and PFLs were included. Exclusion criteria: patients with damage of the ligamentous structures of the medial or lateral compartment due to trauma or to previous surgeries, patients with PFLs and retinacula not discernible due to trauma were excluded.

The tissue samples were preserved in 10% formalin solution and in a refrigerator at 4 °C before the inclusion protocol. The protocol was dehydration of the sample through multiple steps of increasing concentrations of alcohol and xylol, embedding in paraffin and cutting into slices of 6 µm. The tissue was laid on a microscope slide and brought to 37 °C for 12 h in a heater. The slide was then coloured with haematoxylin and antibody anti-S100 (S100 stains Schwann cells forming myelin). All samples were observed with an optical microscope (Leica DC200, 20X zoom) and photographed in colour; fibres and corpuscles were numbered by the operator, and the sample area was measured with ImageJ software. With these two measurements, it was possible to calculate the density of nerve fibres in different tissues. Some slides were coloured with the Weigert–Van Gieson staining for elastic fibres. A total of 50 slides with colouration for nerve fibres and 21 slides with colouration for elastic fibres were examined. The stained slides were analysed by two independent examiners at different times, in a double-blind study.

### 2.3. Radiological Study

The MRIs were selected by searching in our institution database from January 2015 to December 2020. All of the MRIs with evident lesions to the stabilisers of the patella were excluded (acute patellar dislocations, fracture-dislocation of the knee and sports trauma with multi-ligamentous lesions); in all of these cases, there was abundant articular fluid, distension and thickening of the capsular-ligamentous structures [24,25,26,27,28]. All of the MRIs where medial and lateral structures were not identifiable because there were complete ligamentous ruptures or tumour-related alterations were excluded. All of the imaging examinations were performed with a 1.5 Tesla Magnetom avanto (Siemens Healthineers) and a 16-channel phased array knee coil (Siemens). MRIs were without contrast and with 4 mm slices. The MedStation program was used for the MRI measurements, which allowed for electronically computer-assisted retrieval.

One hundred MRIs (96 patients) were selected. The characteristics of patient samples were 52 left knees, 48 right knees. Mean age was 46 ± 19 years (range 16–88), 52 females, 44 males. Patients were enrolled for MRI for knee sprain trauma (S, 33 patients), osteoarthritis (OA, 26 patients), anterior knee compartment pain (AP, 18 patients), tumour (not in the knee), MRI performed for follow-up of disease (T, 14 patients), and other causes—proximal tibia fracture, knee contusions, knee pain, (O, 9 patients). The MRIs where tumour or trauma alterations disrupted the integrity or involved the patellofemoral ligaments and retinacula were excluded according to the selection criteria listed above. MRIs of knees were analysed for the capsular-ligamentous structures and evaluated to understand if retinacula and PFLs were discernible. The measurements of length, width, thickness and insertion of ligamentous structures and the thickness of retinacula and PFLs were studied. For the retinacula, only the thickness was evaluated because they are fascial reinforcements, and it is consequently impossible to define a clear limit for them. The measurements of the patella were obtained from the MRIs. PFLs were measured along scattered lines in different slices following their curved shapes. 

One examiner carried out all of the cadaveric dissections under the supervision of an expert dissector; another independent examiner performed all radiological measurements. 

### 2.4. Statistical Analysis

Data were analysed using Prism 8 (version 8.4.2) Graph Pad Software (GraphPad Software, San Diego, CA, USA). Data are presented as mean ± standard deviation and minimum–maximum. 

The data were checked for normality of distribution using the Shapiro–Wilk test. Differences among means were tested using Student’s t test for normally distributed data, and the Mann–Whitney test and the Wilcoxon matched-pairs signed rank test for non-normally distributed data. Statistical significance was set at *p* < 0.05. 

## 3. Results

### 3.1. Macroscopic Study

In the anterior region of the thigh, the fascia lata covers the muscle bellies of the quadriceps, reaches the patella and goes over it, and continues with the crural fascia in the anterior part of the leg. In the patellar region, the fascia lata finds interdigitations with the periosteum, peritenon, quadricipital aponeurosis and capsular structures of the deep layers. These interdigitations make it difficult to separate the fascia lata from the deep structures.

In the medial region, the fascia is thinner; many muscle fibres of the vastus medialis insert into its inner side. The fascia runs distal to the insertion of the vastus medialis on the proximal-medial corner of the patella, becoming wider and with a crisscrossed texture of fibres formed by fibres coming from the posterior fascia of the sartorius muscle. This part of the fascia, which is wider and crisscrossed, is the medial retinaculum. The medial retinaculum continues on the medial leg surface, becoming thicker due to the deep interdigitations with the distal insertion of the sartorius muscle and fibres coming from the posterior region of the leg and continuing with the ischiocrural tendons. At the pes anserinus level, the fascia is quite adherent, less elastic and fixed to the tendons’ insertions of the pes anserinus.

**Figure 1:**
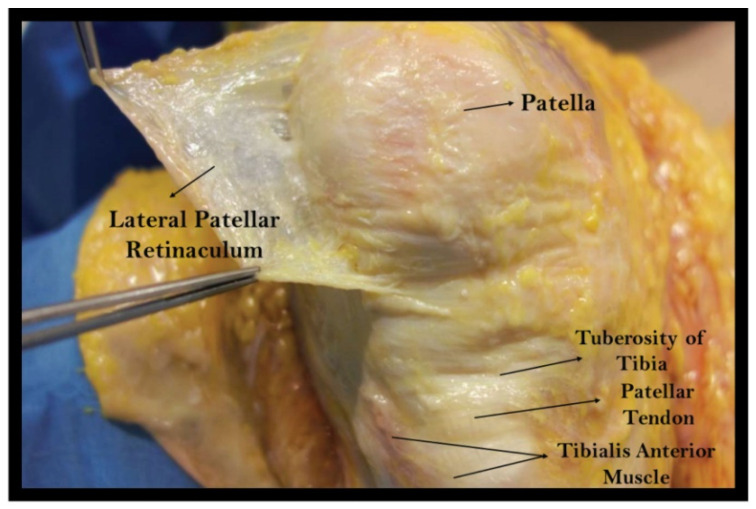
Anatomical study of a right knee, excision of the lateral retinaculum from the antero-lateral aspect of the knee.

In the lateral region, the fascia lata covers the vastus lateralis muscle up to the insertion on the patellar proximal-lateral angle. It then becomes gradually thicker and more adherent laterally and distally. Here, it finds the fibrous thickening of the ilio-tibial tract, covering and partly joining it. Interdigitations between the fascia lata and ilio-tibial tract at the knee level form a crisscross of longitudinal and oblique fibres; this mash of fibres is the lateral retinaculum (Figure 1). This retinaculum has two main systems of fibres: one with longitudinal direction to the Gerdy tubercle (joining then the crural fascia) and one that aims anteriorly to the patella margin and patellar ligament, joining the fascial fibres anteriorly [8,9,12]. 

After cutting the fascia lata and knee retinaculum, this fascial layer could be easily removed both from the medial and lateral compartment.

Once the superficial layer of fascia and retinaculum was removed, the Warren and Marshall [1] second layer of structure was visible, the MCL with a superficial component of fibres going longitudinally from the femoral condyle to the medial surface of the tibia. Slightly posteriorly, there was the muscular belly of the sartorius that divaricated posteriorly, allowing visualisation of the POL. 

From the epicondylar region, in a slightly deeper layer and with interdigitation with the superficial component of the MCL, a bundle of fibres was found with a transversal direction toward the superior-medial angle of the patella and to the medial patellar margin. This bundle of fibres was separable by smooth dissection from the capsular layer, and it was made of fibres with parallel direction diverging only at the insertion on the patellar margin. This bundle is the MPFL [1,2,7,10,11,13], which was found in all the knees in our study. In six knees (four cadavers), lateral insertion of the ligament involved a superior-medial angle and 2/3 of the medial patellar margin, whereas in two knees (one cadaver), the insertion was limited to the proximal 1/3 of the medial patellar margin.

The medial insertion of the MPFL was 1 cm^2^ between the medial femoral epicondyle and the abductor tubercle. In one knee, this insertion was found slightly anterior and proximal to the medial epicondyle. 

The mean length of the MPFL was 48 mm (range 40–58 mm), and the mean thickness at the median third was 14 mm (range 13–16 mm). 

Moving distally to the pes anserinus region and divaricating posteriorly, the wide tendon insertion of the sartorius and tendons of the gracilis and semintendinosus muscles, the wide tibial insertion of the superficial MCL component was found. The insertion was slightly posterior to the insertion of the pes anserinus to which it was connected by fibrous bundles. Further, the patella-tibial and the patella-meniscal ligaments were not identified as clearly distinguishable structures during the dissections.

The dissection was then conducted in the lateral compartment; the lateral retinaculum was removed. The fascia was removed with difficulty from the superior-lateral angle due to myofascial connection with the vastus lateralis muscle. The lateral retinaculum was easily separated from the deep layers along all of the patellar margin with the exception of the triangular part with sides along the medial margin of the lateral femoral condyle (similarly to what was described for the medial compartment), the lateral margin of patellar ligament and the lateral tibial plateau. This point of minor resistance where the capsule and retinaculum are fused together is the point of the antero-lateral arthroscopic portal.

Moving laterally, the retinaculum thickens, acquiring fibres from the iliotibial tract. The iliotibial tract had to be dissected to expose the underlying structures of the deep lateral ligamentous compartment. Here lies the muscular belly of the biceps femoris muscle with its tendinous insertion to the fibular head. Divaricating laterally the biceps femoris belly and divaricating medially the vastus lateralis belly, the cord-like lateral collateral ligament (LCL) that goes toward the insertion on the anterolateral aspect of the fibular head from the lateral epicondyle was visible. From the same area of the epicondyle, antero-distally to the bone pike, there was a bundle of parallel fibres, separable from the capsular layer, inserting at the lateral margin of the patella; this is the LPFL [9,15]. In our study, there was an insertion on the superior 2/3 of the patellar margin in three knees (two cadavers), whereas five knees (three cadavers) presented an insertion in the middle 1/3 of the patellar margin. The mean length of the LPFL was 43 mm (range 35–56 mm) and the mean width was 11 mm (range 9–14 mm) (Table 1).

### 3.2. Microscopic Study

**Figure 2:**
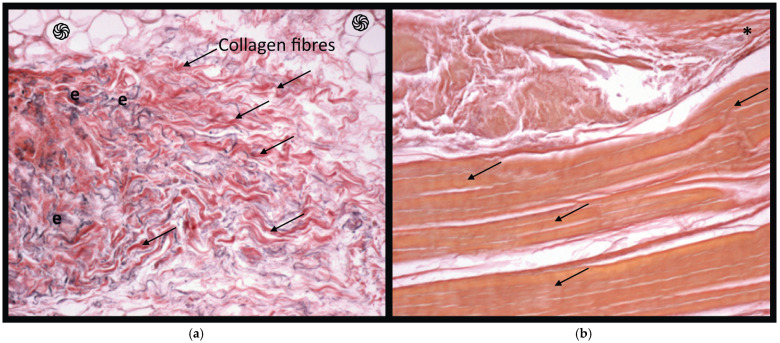
Weigert–Van Gieson staining for elastic fibres used for (**a**) retinacula and (**b**) patellofemoral ligament (10×) biopsy sections, respectively. By this colouration technique, it was possible to identify bundles of collagen fibres (arrows), adipocytes (֍ ), elastic fibres (in violet, e), fibrocytes and small blood vessels (*).

For both the retinacula and PFLs biopsy sections, the haematoxylin + anti-S100 antibody and Weigert–Van Gieson staining for elastic fibres was used (Figure 2). With this colouration technique, connective tissue with bundles of collagen fibres, fibrocytes and small blood vessels were identified. In the retinacula biopsy sections (Figure 2a), there were numerous elastic fibres in blood vessel walls and in the fibrous tissue.

In the ligament biopsy sections (Figure 2b), connective tissue with dense collagen fibres was well represented. Bundles were wider and small blood vessels were found between the bundles of collagen. Elastic fibres were less represented and only a few were inside the collagen bundles (Table 2).

There was a higher density of nerve fibres in retinacula compared to ligaments (*p* = 0.0034). There was also a statistically significant difference in the density of elastic fibres, with higher content of elastic fibres in retinacula (*p* < 0.0005) (Table 3).

**Figure 3:**
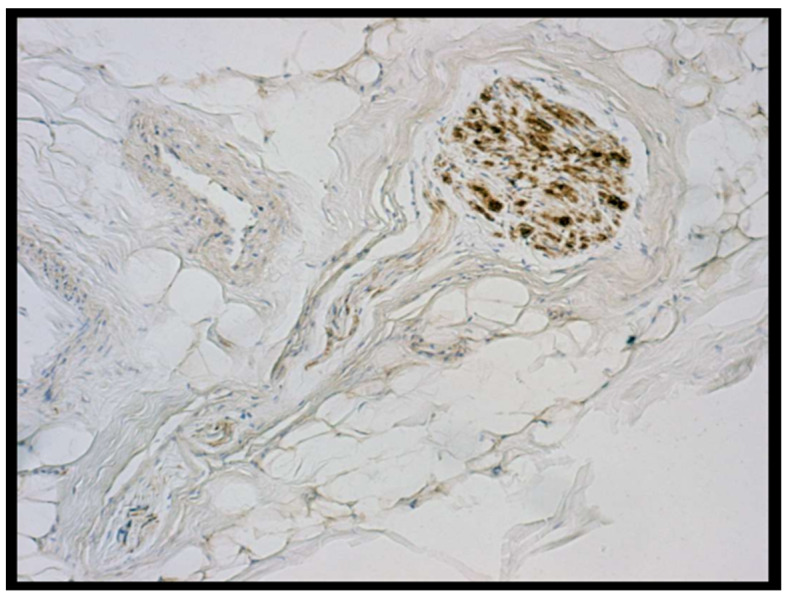
Haematoxylin and antibody anti-S100 colouration. Medial retinaculum section, a Ruffini’s corpuscle (10×).

In the retinacula among nerve fibres, the most represented were free nerve fibres. There were then sparse Ruffini’s corpuscles (Figure 3) and small- to middle-calibre fibres surrounding the paths of small vessels.

### 3.3. Radiological Study

**Figure 4:**
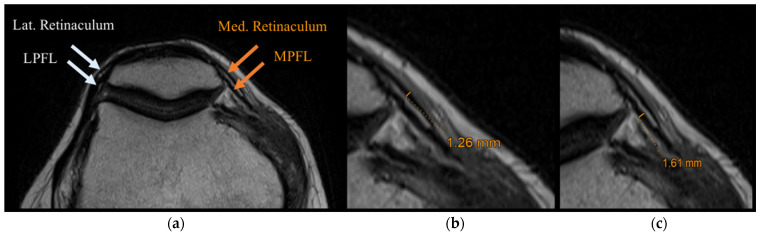
Radiological study: (**a**) MRI with MPFL, medial retinaculum, LPFL, lateral retinaculum indicated; (**b**) measurement of the medial retinaculum; (**c**) measurement of the medial patellofemoral ligament.

In all of the 100 MRIs studied, it was possible to measure MPFL and LPFL lengths. The MPFL length was 50.12 ± 4.14 mm, whereas the LPFL length was 47.50 ± 3.60 mm. When the LPFLs were analysed paired with MPFLs, the LPFL was significantly shorter than the MPFL (*p* < 0.0001) in all groups (S, OA, AP, T). Patella dimensions were mean length of 43.62 ± 4.40 mm and mean width of 44.51 ± 4.24 mm. Only in 32 cases (32%) of all groups (in particular in the groups of knee sprain trauma and anterior compartment pain), the retinacula were clearly discernible from PFLs. In these MRIs, it was possible to measure the thickness of the two structures (Figure 4).

In these cases, the measurement of the mean thickness of the MPFL was 1.40 ± 0.40 mm, and the mean thickness of the LPFL was 1.34 ± 0.33 mm, confirming a statistically significant difference for the length of these structures (*p* = 0.0213). The mean thickness of the lateral retinaculum was 1.40 ± 0.21 mm and that of the medial was 1.32 ± 0.23 mm (*p* > 0.05) (Table 4).

In the OA group, the retinacula and the PFLs were not distinguishable, whereas the retinacula and PFLs were well identifiable in older patients of the S group. There was no difference between medial and lateral retinacula thickness (*p* > 0.05).

## 4. Discussion

This study confirms the three-layer model both for the medial and lateral compartments of the knee joint.

In the medial compartment, the superficial layer is composed of the medial retinaculum, as described by Warren and Marshall [1]. It is a continuation of the fascia lata enveloping the vastus medialis and the sartorius, and towards the pes anserinus adhering to ischiocrural muscle fasciae. In the intermediate layer, there is the MCL, the POL (a posterior thickening of the MCL going to the posterior-medial surface of the proximal tibia) and the MPFL (reinforcing the MCL posteriorly) [13,29]. The MPFL is a different structure and divided from the medial retinaculum and from the joint capsule; it is a parallel fibre bundle [1,2,7,10,11,13]. In contrast to the findings reported by previous authors [14,30,31], the MPFL was found in all of the knees in our study. The medial insertion of the MPFL was between the medial femoral epicondyle and the abductor tubercle. However, in one case, as described by previous authors [10,13,32,33], this insertion was slightly anterior and proximal to the medial epicondyle.

The deep layer of the medial compartment consists of the deep part of the MCL and the joint capsule. An important finding of this study is that the fascia lata, retinacula and quadriceps aponeurosis are indistinguishable in the anterior part of the rotula due to the high number of interconnections. In the posterior part of the medial compartment, the deep and intermediate layers become indivisible, whereas the retinaculum is still distinguishable as an independent structure. The mean length of the MPFL was 48 mm (range 40–58 mm); the thickness at the median third was 14 mm (range 13–16 mm). The mean length of the LPFL was 43 mm (range 35–56 mm), and the mean thickness was 11 mm range (9–14 mm; the measurements were in line with those reported in the literature) [6,10,11,30,34]. In contrast to the findings of other authors [35], in all examined cadavers, there was not a direct insertion of the vastus medialis muscle on the MPFL, although some fibrous expansions from the distal tendinous insertion of the muscle went to the lateral margins of the MPFL. In the medial epicondyle, we found the insertion of the MCL, a wide insertion on all of the medial part of the epicondyle and slightly posteriorly.

There is not a conclusive consensus regarding the description of the lateral compartment of the knee. In this anatomical study, the lateral compartment can be described by a three-layer model: the superficial layer, lateral retinaculum (continuation of the fascia lata enveloping the vastus lateralis); the intermediate layer, made up of the LCL and LPFL (this was a distinguishable structure in all of the knees studied); the deep layer, comprising the joint capsule. The LPFL is recognised by some authors as more of a thickening of the capsule than an autonomous entity. In our study, all of the knees examined presented a clearly distinguishable LPFL, separable from the capsular layer [9,12,14,36]. Regarding the lateral knee retinaculum, as described in the literature [8,9,12], it was found to have two main systems of fibres: one with a longitudinal direction to the Gerdy tubercle and one that aims anteriorly to the patella margin and patellar ligament, joining the fascial fibres anteriorly.

In the histological study, we found a higher density of nerve fibres in retinacula, and the tissue with higher concentration of nerve fibres is the medial retinaculum followed by the lateral retinaculum, the LPFL and the MPFL. The retinacula can have an important proprioceptive role with a high number of free nerve fibres and sparse Ruffini’s corpuscle (mechanoreceptors); this can explain the higher concentration of nerve fibres found by other authors [37,38,39]. Ligaments are static bone stabilisers made of fibrous connective tissue rich in collagen fibres. There are small-calibre nerve fibres, free nerve endings and sparse Ruffini’s corpuscles.

With the Weigert–Van Gieson staining for elastic fibres, we observed a higher percentage of elastic fibres in retinacula compared to ligaments. This was expected because fascial tissue must be elastic to adapt to muscular contraction and different muscular trophism. Retinacula contribute in this way to the dynamic stabilisation of the patella, being influenced by the muscular contraction status [40]. The higher presence of elastic fibres in the medial retinaculum compared to the lateral one, although not statistically significant, can be explained by the different origins of the two retinacula, the lateral one originating from the strong tendon of the ileo-tibial tract. In the MPFL and LPFL, the elastic fibre concentration is lower, whereas the quantity of collagen fibres is higher. This means that ligaments are stronger but less elastic than the retinacula. The results may explain the high rate of medial knee pain after MPFL repair surgery in lateral patella dislocation, when a repair that is too stiff can lead to a suboptimal function of the retinaculum if not appropriately identified [20,21,22].

From the statistical analysis, there was a significant difference in the percentage of elastic and nerve fibres in retinacula and ligaments. This offers support for an anatomical distinction between retinacula and ligaments.

In the imaging studies on the 100 knee MRIs selected and analysed, it was possible to distinguish MPFL and LPFL from retinacula in 32 cases. In sprain trauma of the knee, the retinacula were more identifiable and thicker in older patients, whereas retinacula were thinner in older patients without knee trauma. This fact was explained with the consideration that in the sprain trauma of the knee, there was much more peri-retinacular oedema in older patients.

From a clinical point of view, the results of the present study can be relevant in several ways, and also impact the orthopaedic surgeon’s routine practice. The clear distinction of retinacula, LPFL and MPFL in each knee as different structures of the complex patella stabilisation system is important not only in the cadaver, but also in vivo by MRI. If these structures as described according to the three-layer model for both lateral and medial compartment and their injuries are recognisable by MRI after trauma, an additional diagnostic element can be provided to the surgeons for proper management. In particular, in the case of patella dislocation, patella instability, pathologies involving the patellofemoral joint and even in total knee replacement, the important role of each structure must be considered for planning surgical treatment [41,42].

It is known that the MPFL is biomechanically the most important soft tissue to prevent the patellar from lateral displacement, whereas the contribution of the lateral retinaculum to patella lateral stability is only 10% [43,44,45,46,47]. It has been proven that the injury of MPFL happens in all lateral patellar dislocations [48,49], associated with chondral damages [50]. If the lesion is recognized in its real entity, the surgeon can decide whether to suggest a conservative treatment or perform an operative MPFL re-tensioning or re-construction.

Although multiple MPFL reconstruction techniques have been described, the ideal surgical procedure for MPFL on the patellar side remains controversial [41], and the ideal stiffness after MPFL reconstruction remains unclear [51,52]. Although several studies have indicated that combined MPFL reconstruction with trochleoplasty can result in good postoperative stability and patient satisfaction [53,54], many concerns have been raised about the complication rate and the risk to the cartilage and progression of arthritis in the patellofemoral joint after this procedure [55,56].

Lateral retinacular release has also been reported as a useful treatment for patellar inability. In patellar dislocation, medial patellar ligament injury and quadriceps femoris weakness often occur, which lead to LCL contracture and excessive tension [57]. For these reasons, lateral retinaculum release may be an alternative solution to maintain the stability of the patella. However, its main complication of lateral retinaculum release is LCL patellar medial dislocation or subluxation.

To avoid the several sequalae reported after these procedures, patients with patella instability should be subjected to less invasive interventions focused on reconstruction or retention of only the specific ligamentous structures implicated in the injuries. Obviously, which structure must be reconstructed and when to associate the release must be determined based on MRI images, highlighting the real damage of each structure, which our research has helped to identify, not only for anatomists and radiologists, but also for clinicians and surgeons.

The main limitation of this study is that macroscopic, microscopic and radiological analyses were performed on different groups of patients with difficulties related to the low number of specimens available (eight cadavers for the anatomical study and nine specimens for the histological study) for the cadaveric study design. Further, a histological study bias can be related to the possibility that the specimen analysis gave a different density of elastic and nerve fibres because it was harvested in a specific, operator-dependent site for retinacula and PFLs. Finally, the two main limitations related to the imaging part of the study were as follows. First, the radiographic measurements were conducted on 2D slices of MRIs, which could have caused underestimation of the real measurements; however, multiple measurements at different levels were taken on the same MRI and a mean value was obtained to ensure accuracy. Second, the imaging studies to define normal anatomy were carried out on patients with other pathologies. Hence, future studies should select MRIs of healthy volunteers.

A major strength of this study is that the dissection of the cadaveric specimen was conducted under the supervision of an expert anatomist (C.S.) and on fresh specimens, granting reliable measurements.

Multicentre studies could add value to the basepoint given by this study, increasing the number of specimens and measurements and better defining the role of retinacula and PFL in anterior knee pain.

## 5. Conclusions

The most important findings of this study were that it was possible to confirm that both the lateral and medial compartment can be described using the three-layer scheme, in agreement with other authors. Patellofemoral ligaments and retinacula are separate structures macroscopically and in imaging. The LPFL is separate from the articular capsule layer and is part of the middle layer. The retinacula respond to the higher and specific functional request with a higher nerve fibre content and higher number of elastic fibres compared to the ligaments. The higher number of nerve fibres may indicate a role of the retinacula in anterior knee pain. In order to confirm the results of our study, other investigations with a higher number of patients in the sample are required.

## Figures and Tables

**Table 1:** Measurements from cadaveric dissections.

		Mean (mm)	Standard Deviation	Min–Max (mm)
MPFL	Length	48	5.92	40–58
	Width	14	1.28	13–16
LPFL	Length	43	6.80	35–56
	Width	11	1.67	9–14
PATELLA	Length	53	5.53	40–60
	Width	54	5.53	42–62

(MPFL: Medial patellofemoral ligament; LPFL: Lateral patellofemoral ligament; Min: minimum; Max: maximum).

**Table 2:** Density and number of nerve fibres in ligaments and retinacula.

	Nr	FNE (Mean)	Ruffini Corp (Mean)	TOT Nerves (Mean)	Area (cm^2^) (Mean)	Density (Nr Fibres/cm^2^) (Mean)
TOT	50	5.30	0.30	18.70	1.49	15.60
RET M	17	5.06	0.24	20.24	1.49	18.94
RET L	12	6.33	0.17	20.25	1.41	14.83
MPFL	14	5.64	1.24	17.79	1.48	14.43
LPFL	7	3.43	0.29	14.14	1.65	10.86
Retinacula	29	5.58	0.21	20.24	1.46	17.24
Ligaments	21	4.90	0.43	16.57	1.53	15.34

(FNE: free nerve endings; RET M: medial retinaculum; RET L: Lateral retinaculum; MPFL: Medial patellofemoral ligament; LPFL: Lateral patellofemoral ligament).

**Table 3:** Density of elastic fibres in retinacula and ligaments.

	Number	ELASTIC FIBRES (%)
TOT	21	3.03
M RET	7	4.41
L RET	5	3.59
MPFL	6	1.57
LPFL	3	1.81
Retinacula	12	4.07
Ligaments	9	1.65

(RET M: medial retinaculum; RET L: Lateral retinaculum; MPFL: Medial patellofemoral ligament; LPFL: Lateral patellofemoral ligament).

**Table 4:** Radiological measurements and statistical analysis of the radiological data.

	MPFL Length	MPFL Thickness	MED RET Thickness	LPFL Length	LPFL Thickness	LAT RET Thickness	Patella Length	Patella Width
Number of values	32	32	32	32	32	32	32	32
Minimum	43	0.8	0.8	42	0.57	0.89	37	38
Maximum	64	2.6	1.79	57	2.03	2.1	55	57
Range	21	1.8	0.99	15	1.46	1.21	18	19
Mean	50.12 *	1.40	1.32	47.50 *	1.34	1.40	43.62	44.51
Std. Deviation	4.14	0.40	0.23	3.60	0.33	0.21	4.40	4.24

Length, thickness, and width of MPFL, LPFL, medial (MED RET) and lateral retinaculum (LAT RET). Length and width of the patella. All measurements are expressed in millimetres. (*) statistically significant difference.

## Data Availability

The data presented in this study are available on request from the corresponding author.
